# Staining of Platyhelminthes by herbal dyes: An eco-friendly technique for the taxonomist

**DOI:** 10.14202/vetworld.2015.1321-1325

**Published:** 2015-11-22

**Authors:** Niranjan Kumar, Jadav Mehul, Bhupamani Das, J. B. Solanki

**Affiliations:** Department of Parasitology, Vanbandhu Veterinary College, Navsari Agricultural University, Navsari - 396 450, Gujarat, India

**Keywords:** herbal dyes, Platyhelminthes, rose, sugar beet

## Abstract

**Aim:**

An environment compatible technique to stain Platyhelminthes, *Fasciola gigantica, Gastrothylax crumenifer, Taenia solium*, and *Moniezia expansa* using aqueous and alcoholic extract of sugar beet (*Beta vulgaris*), China rose (*Hibiscus rosa-sinensis*), and red rose (*Rosa hybrida*) were described to minimized the deleterious effects of the synthetic dyes.

**Materials and Methods:**

Aqueous/ethanolic extracts of roses were extracted from the flowers while red beet was extracted from the roots.

**Results:**

Stained helminthes acquired a comparable level of pigmentation with the distinction of their internal structure in these natural dyes. The flukes (liver and rumen) internal structure, oral and ventral/posterior sucker, cirrus sac, gravid uterus, testes, ovary, and vitallaria were appeared pink color in aqueous and alcoholic extract of either China or red rose and yellow to brown color in sugar beet stain. The interior of the proglottid of *T. solium* and *M. expansa* took yellow to brown color with good contrast in sugar beet stain and of pink to pink-red in China and red rose stain.

**Conclusion:**

The extract of roses (red rose followed by China rose) followed by red beet possess the potential to replace the conventional stains in the taxonomic study of Platyhelminthes parasites.

## Introduction

The effective and sustainable level of control of parasites depends on their specific detection. In parasitological laboratory, the more accurate identification of parasite, especially in taxonomic studies of trematode, cestode, nematode, and also for the differentiation of parasitic egg and larva is still based on the detection of specific morphological features and details of internal structures after staining them using synthetic/natural dyes, hematoxylin, eosin, Romanowsky stain, Lactophenol cotton blue, Lugol’s iodine, malachite green, carmine, etc. [1].

The most widely used hematoxylin, a natural dye extracted from the wood of the logwood tree (*Hematoxylon campechianum*) found in Mexico and Central America which after oxidation achieve staining ability due to the formation of cationic hematein-Al^+3^ molecule [2]. Eosin is a synthetic dye, a member of the xanthene family of dyes, derived from fluorescein. Its acidic negatively charged features enable it to bind with positively charged proteins (cationic proteins) - Arginine, lysine, and histidine at lower pH in the cytoplasm, connective tissue, and nuclear proteins. Alcoholic eosin Y formulated to produce optimal contrast with hematoxylin which stains erythrocytes bright red/pink and collagen, muscle and cytoplasm varying shades of orange/pink [3].

However, these conventional dyes are imported and expensive material and some of them can cause an allergic reaction, irritation and burning sensation to eye, skin, nose, and throat while some are of carcinogenic in nature [4]. In addition to that, exposure of high concentration of iodine vapor may results in airway spasm, chest tightness, breathing difficulty, severe inflammation and fluid accumulation in the voice box, upper airways, and lungs [4]. Therefore, the use of herbal dyes is an effective and safe alternative.

Isolating herbal dye from extracts of leaves, flower, bark, root, and other parts of some ornamental plant species mostly involve powdering, mixing with other materials, boiling in water, and dissolving in inorganic or organic solvent [5]. Until the late nineteenth century the natural dyes extracted from plants, animals, and minerals were used as primary staining material in the textile, paint, and other industries [6]. But, with the pace of development of synthetic dyes as it is cheaper, brighter, more colors, fast and easy to use, the use of natural dyes have decreased [7]. As the public becomes aware of ecological and environmental problems related with the synthetic dyes the use of natural dyes has once again gained interest [8]. Natural dyes can stand as a much-needed alternative to the complex world of chemical dyes [9]. Although methods of preparation of herbal dyes are more complex than the commercial dyes but their staining quality and stability are far better than the synthetic dyes [6]. Vibrant colors can be produced from natural dyes by mixing them with each others in different proportion. Furthermore, these natural dyes could consequently provide a new economy source to the country.

The sugar or red beet (*Beta vulgaris*) plant belongs to subfamily Betoideae of Amaranthaceae family with basal leaves forming a rosette and the roots are stout, sometimes conspicuously swollen forming a beet together with the hypocotyls [10]. The roots are enriched in amino acids, sucrose, minerals, vitamins, and dyes [11]. They can be used in many ways, as medicine in various ailments and importantly as a natural colorant which include yellow, orange, red, and purple colors in ice cream, beverages, and some fruit products [12,13].

The China rose, *Hibiscus rosa-sinensis*, an evergreen herbaceous ornamental plant belongs to family malvacea, native to East Asia and is grown throughout the tropical and subtropical region of the world [14]. It has a number of medicinal uses, as it contains polyphenols, flavonoid, and anthocyanins. It may have some potential in cosmetic skin care as an anti-solar agent by absorbing ultraviolet radiation [15]. It can also be used as a colorant to various edible and in-edible items [13].

The genus *Rosa*, belongs to Rosaceae family, contain over 100 species and 1000 of cultivars and hybrids. It has colorful flowers ranging from white through yellows and reds. Most species are native to Asia, with smaller numbers native to Europe, North America, and Northwest Africa. Roses are best known as ornamental plants grown for their flowers in the garden through the world. They have been also used for commercial perfumery and commercial cut flower crops. They are made into syrup, jam, jelly, marmalade, and soup or are brewed for tea, primarily for their high Vitamin C content. It can be used in skin products and some makeup products. Rose water due to its distinctive flavor is used in sweets. They also have minor medicinal uses [16].

This study was planned to evaluate the effectiveness of aqueous and alcoholic extracts of herbal dyes such as extract of red beet (*B. vulgaris*), China rose (*H. rosa-sinensis*), and red rose (*Rosa hybrida*) as an alternative stain to conventional dye, hematoxylin in the staining of a flatworms.

## Materials and Methods

### Ethical approval

Not required, as the experiment was done using plant extracts and the parasites collected during post-mortem examination.

### Collection and preparation of plants materials

Flowers were collected from the university garden while the roots of red beet were purchased from the local market. Petals of the flowers were separated by hand picking, and red beets were sliced into small piece.

### Double distilled water extraction

Fifty gram of the undesiccated plant materials were mixed with 100 ml (0.5 g/ml) of double distilled water and boiled for half an hour in a microwave oven. After cooling the extracts were proceeded to purification procedure.

### Ethanol extraction

Fifty gram of undesiccated plant materials were mixed with 100 ml of 96% ethanol (0.5 g/ml). Then, they were incubated at 4°C for 24-48 h for complete extraction of the stains. The extracts were proceeded to purification procedure.

### Purification of extract

At the end of each extraction procedure, the extracts were purified by a two steps filtration process. Initially, the extract was filter through wire mesh followed by filtration using Whatman filter paper. Filtrates were centrifuged at 5000 rpm for 30 min. The supernatant was collected into a reagent bottle and after proper labeling stored at 4°C until next usage.

### Parasites specimens

The whole or segments of Platyhelminthes, *Fasciola gigantica*, *Gastrothylax crumenifer*, *Taenia solium*, and *Moniezia expansa* specimen included in this study were collected from departmental museum stored in 10% formalin.

### Staining of the parasite

The whole or segments of Platyhelminthes specimen were tied and fixed between two slides and preserved in 10% formalin. After fixation and flattening the specimens were transferred from 10% formalin into the 70% ethanol for at least 15 min. The specimens were immersed into the stains solution and incubated for 1-2 days. In order to remove the excessive stain without loss of pigmentation, the specimens were incubated into acid alcohol (2 ml of concentrated HCl in 100 ml of 70% ethanol) for 1-5 min. The destained specimens were again transferred into the 70% ethanol for 1 h to dehydrate them. For further dehydration, the specimens were placed into 80, 90, and 100% ethanol for 1 h in each solution. The dehydrated specimens were clear into the clearing agent, clove oil. After clearing, the specimen was mounted on to the slides using diastrenedibutyl phthalate xylene. Hematoxylin solution and saline mount were used as a control in this study.

## Results

The extracts maintained their dying ability and stability for about 1 month when stored at 4°C. All extracts showed slightly acidic condition, when examined using pH paper. The red beet extracts appeared dark red to brown in color while extracts of the rose appeared pink to pink-red in color.

**Figure-1:**
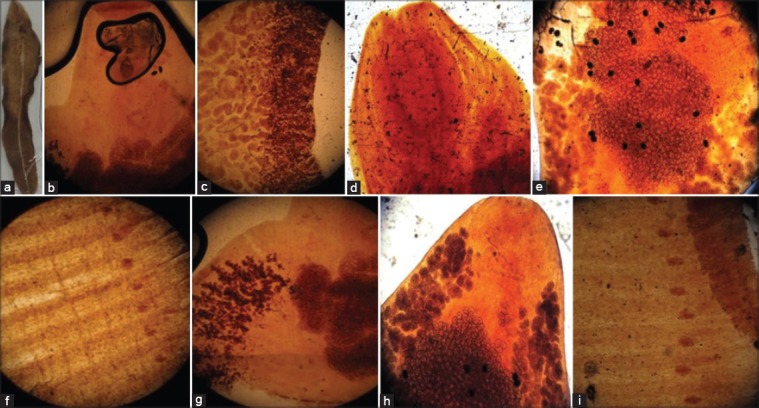
Platyhelminthes stained in extracts of sugar beet (Aqueous extract: (a) *Fasciola gigantica*, (b) anterior part of *F. gigantica*, (c) middle part of *F. gigantica*, (d) anterior part of *Gastrothylax crumenifer*, (e) middle part of *G. crumenifer*, (f) segments of *Moniezia expansa*; alcoholic extract: (g) anterior part of *F. gigantica*, (h) anterior part of *G. crumenifer*, (i) segments of *M. expansa*).

**Figure-2:**
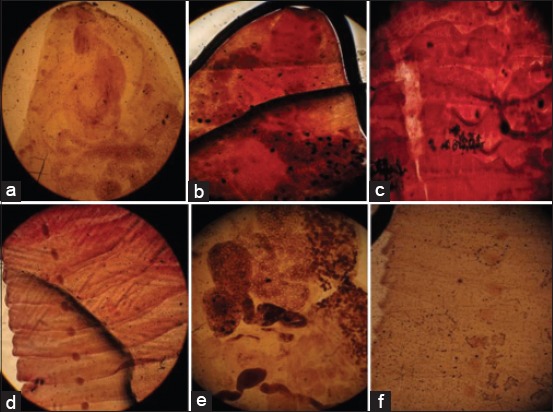
Platyhelminthes stained in extracts of China rose (Aqueous extract: (a) Anterior part of *Fasciola gigantica*, (b) anterior part of *Gastrothylax crumenifer*, (c) segments of *Moniezia expansa*, (d) segments of *Taenia solium*; alcoholic extract: (e) middle part of *F. gigantica*, (f) segments of *T. solium*).

**Figure-3:**
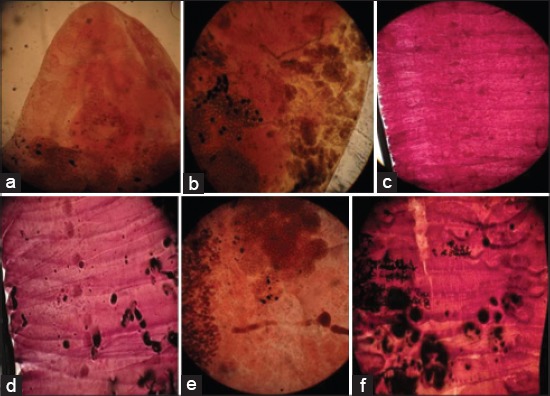
Platyhelminthes stained in extracts of red rose (Aqueous extract: (a) Anterior part of *Fasciola gigantica*, (b) middle part of *Gastrothylax crumenifer*, (c) segments of *Moniezia expansa*, (d) segments of *Taenia solium*; alcoholic extract: (e) middle part of *F. gigantica*, (f) segments of *M. expansa*).

**Figure-4:**
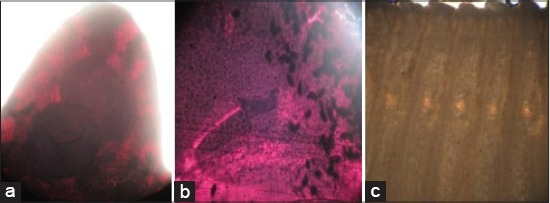
Platyhelminthes stained in: Hematoxylin ((a) Anterior part of *Fasciola gigantica*, (b) middle part of *Gastrothylax crumenifer* stained in hematoxylin solution); saline ((c) segments of *Moniezia expansa*).

Stained helminthes acquired a varying degree of pigmentation with the distinction of their internal structure using these plant extracts as comparable to control (Figures-1-4). The color of the extracts was absorbed by the parasites tissues but the color intensity differs between each extraction method. The tropical liver and rumen fluke’s internal structure, oral and ventral/posterior sucker, cirrus sac, uterus with eggs, testes, ovary, and vitallaria were appeared light to deep pink color in aqueous and alcoholic extract of China and red rose while yellow to brown color in sugar beet extract (Figures 1a-e, g, h; 2a, b, e; and 3a, b, e). Rose’s extracts were better than sugar beet extract in staining the worm, *F*. *gigantica* and *G*. *crumenifer*, especially its sucker, branched intestine, vitelline gland, vitelline duct, ootype, and body spines (Figures-1a-e, g-h; 2a-b, e; and 3a-b, e). The interior of the proglottid (the cirrus, cirrus pouch, and vas deference) and aggregation of eggs of *M. expansa* and *T. solium* took yellow to brown color with good contrast in sugar beet and pink to pink-red in China and red rose stain (Figures-1f, i; 2c-d, f; and 3c-d, f). The methods of extraction do not imparted a too much significant difference in the coloring pattern of the stained specimens. The red rose aqueous/alcoholic extract was a suitable dye and clearly defined the internal organ of the parasites, body surface spines, egg, and especially the branched tests of trematodes as compared to China rose extract (Figures-2 and 3). The aqueous extract of China/red rose imparted more contrasting color than the alcoholic extract as boiling water extracted more and more pigmenting agent than the alcohol (Figures-2 and 3).

All these alternative dyes were able to retain the color in the permanent mount of the Platyhelminthes for more than 6 months (until the drafting of the manuscript).

The set staining protocol yielded over-staining and diffused staining pattern in Harris hematoxylin solution (Figure-4a and b) and saline solution (Figure-4c), respectively.

## Discussion

In order to determine the taxonomic position and identification purposes, the differential staining of Platyhelminthes were tried using various plant extracts as an alternatives to conventional staining procedures [17]. The staining characteristic of *F*. *gigantica*, *G*. *crumenifer*, *M*. *expansa*, and *T*. *solium* is almost same as they belong to the common phylum Platyhelminthes and thus they are histologically same [18].

The pH of the extract determines the ability of a dye to stain specific tissue structures [19]. Acidic and basic structures are stained by basic and acidic dyes, respectively. Acidophilic stains (acidic pH <7) retain a higher concentration of H^+^, thus bind to negatively charged molecules in the tissues (basic tissues). Basophilic stains (basic pH >7) retain lower concentration of H^+^, thus bind to positively charge molecules in the tissues (acidic tissues). Charge distribution of the dye determines attractive or repulsive characteristics of the dye. Cations (+)/positive and anions (−)/negative charges are attracted to negatively and positively charged molecules, respectively. Thus, charge is largely determined by pH of the dye extracts. High pH solution decreases the number of charged groups within the tissue and *viz*. [3]. Thus, the pHs of the extracts could be a reason explaining why these extracts were able to stain Platyhelminthes differential.

Affinity is the results of attractive forces between the dye molecule and molecules within the tissue. Dyes have a greater affinity for tissue molecules than solvent molecules. The affinity of dyes for tissue elements is affected by a number of factors: Structure, shape, and charge distribution of the dye molecule and the solvent characteristics [2].

The sugar beet extracts imparted yellow to brown color to the parasites specimens as they are enriched in many pigment component like betacyanin (red color) and betanin, isobetanin and betanidin (yellow color) [20,21]. The roses imparted pink to pink-red color due to the presence of flavonoids pigments, including most prevalent water-soluble anthocyanin pigment [22]. Okolie [23] listed ethanol as an ideal extractant as it can extract maximum dyes soluble in organic solvents, being volatile in nature highly permeable to the tissues/tegumental coat of the parasites and an important anti-microbial agent. Although in this study the staining quality does not differ significantly as per extraction methods adopted for the extraction of the colorant from the plant materials.

The Platyhelminthes acquire varying degree of pigmentation with the distinction of their internal structure due to variances in their structure, the body or tegument of trematodes and cestodes composed of muscle fibers, layer of glycocalyx and calcareous corpuscles, and other substance [24].

The results of the study evidenced that extract of red rose followed by China rose than red beet possess the potential to replace the conventional dye in staining procedure in the taxonomic study of helminthes parasite. Ethanol extracts of the plant materials could be a good substitute for conventional dyes.

## Authors’ Contributions

NK conceptualized the aim of the study, designed, planned and supervised the experiment, interpreted the results, and drafted and corrected the manuscript. JM and BD authors were actively involved in the execution of the experiment. JBS helped in the analyses, draft, and revision of the manuscript. All authors read and approved the final manuscript.
